# Lateralization discordance between stereo EEG and scalp EEG in temporal epilepsy: A case report

**DOI:** 10.1016/j.ebr.2025.100803

**Published:** 2025-07-05

**Authors:** Spencer Gunnell, Audrey Nath, Patrick Karas, Todd Masel

**Affiliations:** The University of Texas Medical Branch, USA

**Keywords:** Stereo-EEG, Refractory Epilepsy, Scalp EEG, Discordance, Lateralization

## Abstract

A 34-year-old woman with drug-resistant epilepsy underwent simultaneous scalp and stereo-EEG (sEEG) and had seven seizures, three of them without visibility on the scalp EEG. Of the four seizures seen on both sEEG and scalp EEG, the seizure onset in the scalp EEG was ipsilateral to the seizure onset in the sEEG in two seizures and contralateral to the sEEG onset in two seizures. This unusual lateralization discordance of scalp EEG and sEEG highlights the importance of bilateral lead placement in stereo EEG to accurately identify the seizure onset zone.

## Highlights

• Localization of seizure onset zones in drug-resistant epilepsy can be challenging.
• Lateralization of seizure onset on scalp EEG and stereo-EEG can be discordant.
• Stereo-EEG’s value in localization of seizure onset zones is seen in this case report.

## 1 Introduction

Evaluation of scalp EEG and seizure semiology are key initial steps in lateralizing and localizing a seizure onset zone in patients with drug-resistant epilepsy. This initial step of pre-surgical evaluation is performed in The Epilepsy Monitoring Unit (EMU), where video EEG captures both electrographic and clinical features of seizures. These results, combined with tests such as brain MRI, neuropsychological assessments, PET scan, and magnetoencephalography (MEG), are crucial in identifying a seizure onset zone. When there is still ambiguity regarding the location of the seizure onset zone, intracranial EEG monitoring, such as stereo EEG (sEEG), can be performed. In the case of sEEG, results from the phase 1 pre-surgical evaluation are utilized to determine where electrodes are placed. This process assumes these tests, especially scalp EEG, reliably predict lateralization and localization of the seizure onset zone. The following case illustrates significant discordance in lateralization between the scalp EEG and sEEG, challenging this assumption.

## 2 Case Presentation

A 34-year-old right-handed woman with drug-resistant epilepsy began having seizures at the age of 11. She had multiple seizure types, including focal preserved consciousness seizures characterized by intense deja-vu and visual disturbances described as seeing through stained-glass, focal impaired consciousness seizures described as staring spells, and rarely focal-to-bilateral tonic-clonic seizures. Her seizures occurred up to five times a day. She had no family history of seizures, perinatal anoxia, history of febrile convulsions, severe traumatic brain injury, CNS vascular disorders, or CNS neoplastic disorders. Her only risk factor was a history of chickenpox with a high fever at age 6. Physical examination did not reveal any signs of cortical injury. She had already failed six antiseizure medications due to either lack of efficacy or intolerance to the medication. Despite vagal nerve stimulator (VNS) implantation and optimal dosing of valproic acid and lacosamide, she continued to have 5–6 seizures per month.

The patient underwent evaluation for epilepsy surgery due to drug-resistant epilepsy. In two prior EMU studies with a scalp EEG using the 10–20 electrode system, 52 seizures were recorded. Forty-three seizures originated in the left temporal region, and 9 seizures originated in the right temporal region. Of these 52 seizures, 50 were electrographic and 2 were electroclinical. The electroclinical seizures originated in the left temporal region.

A 3 T brain MRI and interictal FDG-PET scan were normal. Neuropsychological evaluation revealed lower visual memory compared to verbal memory, supporting a right temporal seizure onset zone. The results of the neuropsychological evaluation were potentially confounded by previously diagnosed conditions of post-traumatic stress disorder, depression, and anxiety that may have impacted attention and reasoning skills.

**Fig. 1:**
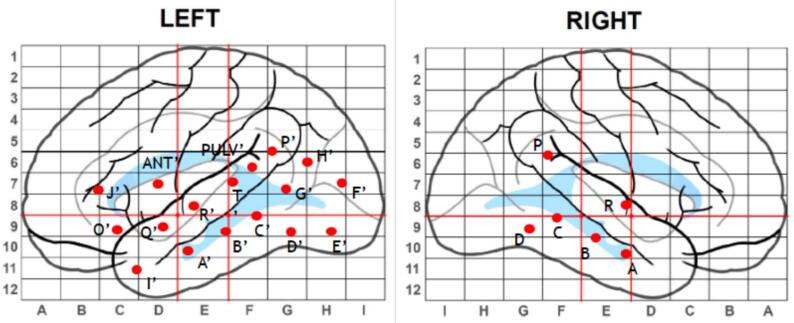
sEEG electrode implantation schematic depicting coverage of the left temporal-parietal-occipital network and right temporal network. Each depth electrode chain is represented by a small red circle, with the label adjacent to it. “ANT” refers to anterior nucleus of the thalamus, and “PULV” refers to the pulvinar. The number of contacts for each lead can be found in the supplementary material.

Based on the results of the EMU studies, imaging studies and neuropsychological assessment, the decision was made to perform sEEG using 23 intracranial electrodes bilaterally: 17 in the left hemisphere, including the left frontal, left temporal, left occipital lobes and left anterior and pulvinar thalamic nuclei, and six in the right temporal lobe (see Fig. 1). The primary hypothesis was left mesial temporal epilepsy. The secondary hypothesis was right temporal epilepsy. Extra leads were placed in the left temporal-parietal-occipital network due to the presence of visual auras. A modified scalp EEG with only bitemporal chains was concurrently placed.

During the ten-day combined intracranial and scalp EEG study, home medications were tapered within three days, and the vagal nerve stimulator device was turned off. Seven seizures were captured, three electrographic (subclinical) and four electroclinical. The latter presented with disorientation and unresponsiveness, non-versive rightward and leftward head turn, oral automatisms, left and right-hand automatisms, right arm extension and left arm flexion, and tonic-clonic activity. The seizure onset was not visible on the scalp EEG for three of the seven seizures. For two of the seizures, the seizure onset in the scalp EEG was ipsilateral to the seizure onset on sEEG. In two other seizures, the seizure onset in the scalp EEG was contralateral to the seizure onset on sEEG (Table 1).

**Table 1:** In the 3rd and 4th columns, “L” refers to a left temporal onset, “R” refers to a right temporal onset, and N/A refers to the absence of scalp EEG seizure.

SEIZURE	CLINICAL SEIZURE	sEEG ONSET	SCALP EEG ONSET	CONCORDANT ON SCALP	DELAY IN ONSET BETWEEN sEEG AND SCALP EEG
1	YES	L	None	N/A	N/A
2	YES	R	L	No	5 s
3	YES	L	L	Yes	30 s
4	YES	L	R	No	51 s
5	NO	R	R	Yes	120 s
6	NO	R	None	N/A	N/A
7	NO	R	None	N/A	N/A

**Fig. 2:**
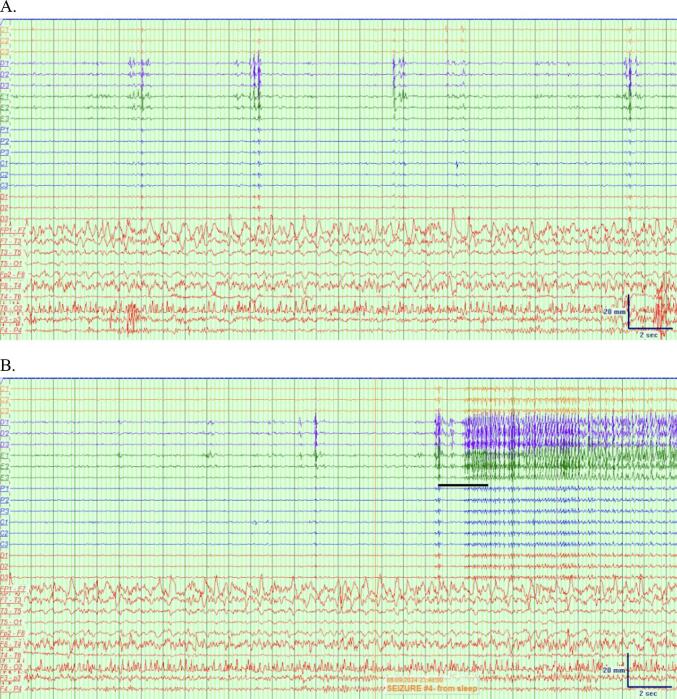

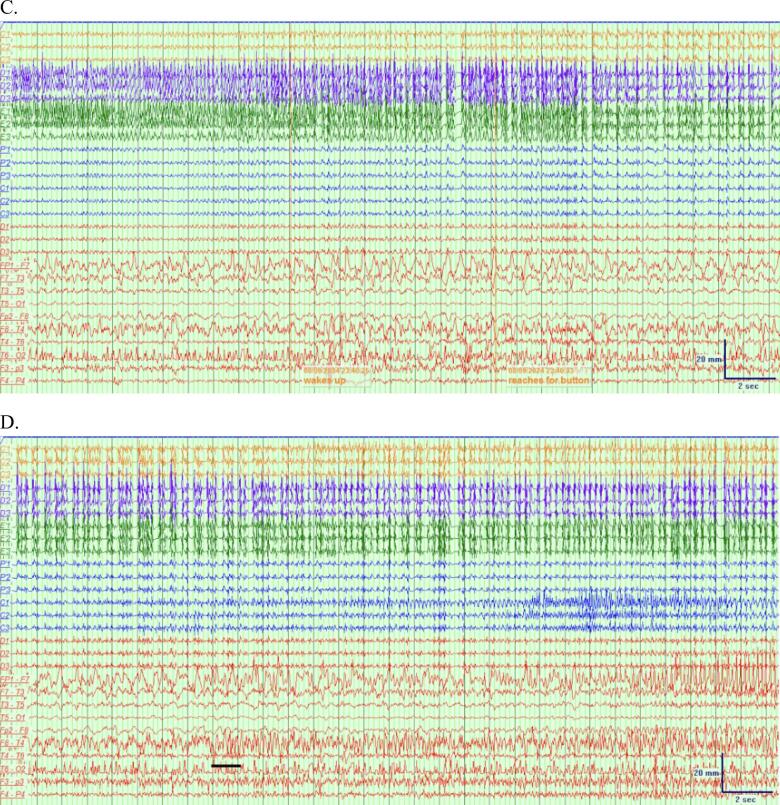
A combined sEEG and scalp EEG montage. Filter settings − sEEG: LFF 5 Hz, HFF 200 Hz, Notch 60 Hz, Sensitivity 200 uV/mm, timebase 15 mm/sec. Scalp EEG: LFF 5 Hz, HFF 30 Hz, Notch 60 Hz, Sensitivity 10 uV/mm, timebase 15 mm/sec. A. The epoch prior to seizure onset on sEEG, showing polymorphic delta activity that does not evolve in Fp1-F7. This activity was intermittently present for most of the recording. B. sEEG electrode tracings at onset of seizure #4 at C’ 2–4. sEEG onset occurred at 23:40:02. C. Evolution of the seizure on sEEG. D. Scalp EEG tracing at onset of seizure #4 characterized by 6–7 Hz sharp waves at F8 and T4 with subsequent 6 Hz sharp waves at Fp1 and F7. Scalp EEG onset occurred at 23:40:53, 51 s after sEEG onset.

An example of the sEEG and scalp EEG onsets in which laterality is discordant is shown in Fig. 2. Fig. 2B shows the sEEG onset for seizure #4, in which there are clear sharp waves maximal in the left mesial temporal region which evolve to adjacent depth electrodes and evolve in frequency to 8 Hz rhythmic spikes. Fig. 2D shows the simultaneous scalp EEG, in which there is evolution of sharply-contoured 6–7 Hz slowing maximal in the right temporal region F8-T4 derivation.

## 3 Discussion

This case of bitemporal epilepsy, recorded with simultaneous sEEG and scalp EEG, demonstrated low concordance in seizure onset lateralization; only two seizures showed concordant laterality, three were not visible on scalp EEG, and two exhibited discordant laterality, with contralateral seizure onset appearing on scalp EEG (Fig. 2). Most studies suggest high lateralization concordance between intracranial EEG and scalp EEG, contrasting this case. One study compared scalp and depth EEG seizure patterns in 34 patients with intractable TLE and found that seizure propagation on scalp EEG during clinical events was typically bilateral, synchronous, or ipsilateral, but not contralateral [1]. A study performing simultaneous intracranial and surface EEG in 24 patients found that 17 of patients exhibited scalp EEG seizures lateralizing to the same side as intracranial EEG [2]. The scalp EEG for the other 7 patients showed a diffuse disruption of the background activity or diffuse slowing, but no epileptiform findings contralateral to the intracranial EEG [2]. In a more recent study, 172 seizures in 27 patients with drug-resistant epilepsy, consisting of individuals with and without lesions on imaging, were captured on sEEG [3]. Scalp EEG was performed simultaneously and captured 100 of the 172 seizures [3]. This study showed that for focal-to-bilateral tonic-clonic seizures and focal impaired consciousness seizures, visibility on scalp EEG was high at 100 % and 97 % respectively, while only 33 % of focal preserved consciousness seizures and subclinical seizures were visible [3]. Interestingly, of the 100 seizures captured on both scalp EEG and sEEG, only one seizure in a patient with polymicrogyria demonstrated discordance in laterality of seizure onset, demonstrating the rarity of this discordance [3]. A study of 129 patients that included patients with and without lesions on brain MRI corroborated the rarity of this finding, with only 3 patients demonstrating discordance in laterality between scalp EEG and intracranial EEG [4].

Additional cases of false lateralization between scalp EEG and intracranial EEG in patients with lesions on brain imaging have been reported in the literature. In a study of five patients with prominent unilateral hippocampal sclerosis, seizures on scalp EEG localized to the contralateral temporal lobe [5]. The authors hypothesized that severe damage to the neocortex of the sclerotic hippocampus impairs neuronal recruitment, delaying detectable scalp discharges until the seizure has propagated to the side contralateral to the hippocampal sclerosis [5]. Intracranial EEG performed in these patients confirmed that seizure onset was ipsilateral to the sclerotic hippocampus [5]. In contrast to the previous examples, our patient had a normal brain MRI. In a similar case of drug-resistant temporal lobe epilepsy with non-lesional imaging, the investigators reported false lateralization on scalp EEG, with all eight recorded seizures localizing to the left temporal lobe [6]. Subsequent ECoG, performed separately, captured five seizures that all originated from the right temporal lobe with rapid propagation (approximately 1.5 s) to the left temporal lobe, showing that the scalp EEG had falsely lateralized the seizure onset zone [6]. The limitation in this case was that scalp EEG and ECoG were not performed simultaneously, unlike our patient.

Another notable finding in our patient was the absence of scalp EEG correlates in three of seven seizures. A previous study suggested that visibility of a seizure on scalp EEG depends on source depth, with higher concordance between sEEG and scalp EEG when the seizure onset zone is superficial rather than deep [7]. Interictal and even ictal discharges confined to mesial temporal structures are often undetectable on scalp EEG [8]. Seizures that do not recruit as much neocortex, such as subclinical seizures and focal preserved consciousness seizures, are less likely to be visible on scalp EEG with electrodes placed according to the 10–20 electrode system [1,3]. In the three seizures not captured on scalp EEG in our patient, two originated in the right temporal lobe with no clear clinical signs. The third seizure originated in the left temporal lobe and was characterized by a non-versive head turn to the right and non-purposeful left-hand movements with brief unresponsiveness to one question asked during the seizure.

Timing is another factor of concordance between scalp EEG and sEEG. A cohort study reported an approximate delay of 10 s in scalp EEG relative to sEEG in 25 % of patients, although recordings were not simultaneous [9]. Only one scalp EEG onset occurred within 10 s of sEEG onset in our patient, with the other three seizures delayed by 30 s, 51 s, and two minutes. This delay in scalp EEG onset may be due to multiple factors, including attenuation by the skull of discharges that are fast and low in amplitude and insufficient recruitment of cortical surface area [9].

There are a few possible reasons for the mismatch in lateralization between sEEG and scalp EEG. The delay between sEEG onset and scalp EEG onset was long enough in duration to allow for the seizure to propagate to the contralateral hemisphere. On sEEG, three of seven seizures originated from the left hemisphere and four from the right hemisphere, suggesting either multifocal seizure onset zones or a seizure onset zone outside the sampled regions. Such discordance has important implications for surgical outcomes. A study of 13 patients undergoing simultaneous scalp EEG and sEEG found that concordance in latency, pattern, and location of seizure onset was associated with better postoperative outcomes, whereas discordance suggested a seizure onset zone in regions not covered by sEEG [10]. Similarly, another study reported that in patients who achieved two years of seizure freedom after temporal lobectomy, late lateralizing features on scalp EEG correctly localized to the resected side in 96 % of seizures. In those with ≥ 90 % seizure reduction, 67 % of such features lateralized to the surgical side [11].

## 4 Conclusion

This is a unique case of a 34-year-old with drug refractory epilepsy and non-lesional imaging where scalp EEG and sEEG were performed simultaneously with significant discordance in lateralization between these two modalities. As seen in this case, scalp EEG and sEEG are essential tools in the comprehensive evaluation of patients with drug-resistant temporal epilepsy. In particular, sEEG played an important role in accurately localizing and lateralizing seizure onset zones, and it was essential for successfully implanting a responsive neurostimulator (RNS) device. Following RNS implantation, our patient experienced a significant reduction in seizure frequency, including several months of no seizures. Discordance in lateralization between sEEG and scalp EEG is a rare finding. This case highlights the importance of extensive sEEG electrode coverage, including bilateral coverage, to decrease the chance of missed seizure onset zones that may have been undetected by scalp EEG.

## CRediT authorship contribution statement

**Spencer Gunnell:** Writing – review & editing, Writing – original draft. **Audrey Nath:** Writing – review & editing, Writing – original draft, Supervision. **Patrick Karas:** Writing – review & editing, Supervision. **Todd Masel:** Writing – review & editing.

## Declaration of competing interest

The authors declare that they have no known competing financial interests or personal relationships that could have appeared to influence the work reported in this paper.
