# Supplementary figures and images for: Lateralization discordance between stereo EEG and scalp EEG in temporal epilepsy: A case report
Source: Epilepsy Behav Rep. 2025 Jul 5;31:100803. doi: 10.1016/j.ebr.2025.100803 (PMC12274905; doi:10.1016/j.ebr.2025.100803)

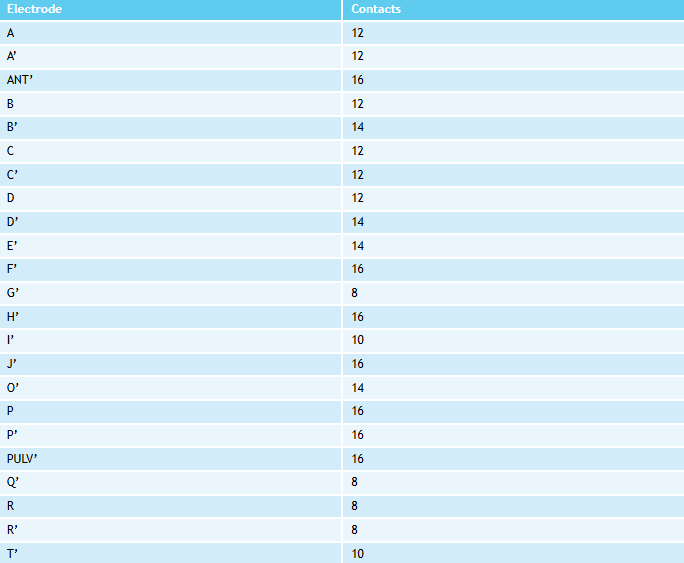

Supplement: Supplementary Data 1 [file mmc1.docx]
